# Tumour draining lymph node-generated CD8 T cells play a role in controlling lung metastases after a primary tumour is removed but not when adjuvant immunotherapy is used

**DOI:** 10.1007/s00262-021-02934-3

**Published:** 2021-04-09

**Authors:** Vanessa S. Fear, Catherine A. Forbes, Samuel A. Neeve, Scott A. Fisher, Jonathan Chee, Jason Waithman, Shao Kang Ma, Richard Lake, Anna K. Nowak, Jenette Creaney, Matthew D. Brown, Christobel Saunders, Bruce W. S. Robinson

**Affiliations:** 1 Institute for Respiratory Health, National Centre for Asbestos Related Diseases, University of Western Australia, Perth, Australia; 2 Telethon Kids Institute, Perth, Australia; 3 Medical School, School of Biomedical Sciences, University of Western Australia, Crawley, WA Australia; 4 Fiona Stanley Hospital, Murdoch, Australia; 5 Division of Surgery, Medical School, University of Western Australia, Perth, Australia

**Keywords:** Cancer surgery, Metastatic disease, Lymph nodes, Immunotherapy

## Abstract

Surgical resection of cancer remains the frontline therapy for millions of patients annually, but post-operative recurrence is common, with a relapse rate of around 45% for non-small cell lung cancer. The tumour draining lymph nodes (dLN) are resected at the time of surgery for staging purposes, and this cannot be a null event for patient survival and future response to immune checkpoint blockade treatment. This project investigates cancer surgery, lymphadenectomy, onset of metastatic disease, and response to immunotherapy in a novel model that closely reflects the clinical setting. In a murine metastatic lung cancer model, primary subcutaneous tumours were resected with associated dLNs remaining intact, completely resected or partially resected. Median survival after surgery was significantly shorter with complete dLN resection at the time of surgery (49 days (95%CI)) compared to when lymph nodes remained intact (> 88 days; *p* < 0.05). Survival was partially restored with incomplete lymph node resection and CD8 T cell dependent. Treatment with aCTLA4 whilst effective against the primary tumour was ineffective for metastatic lung disease. Conversely, aPD-1/aCD40 treatment was effective in both the primary and metastatic disease settings and restored the detrimental effects of complete dLN resection on survival. In this pre-clinical lung metastatic disease model that closely reflects the clinical setting, we observe decreased frequency of survival after complete lymphadenectomy, which was ameliorated with partial lymph node removal or with early administration of aPD-1/aCD40 therapy. These findings have direct relevance to surgical lymph node resection and adjuvant immunotherapy in lung cancer, and perhaps other cancer, patients.

## Introduction

Surgical resection of cancer remains the frontline therapy for some 12 million cancer patients every year [1]. However, surgery alone is unlikely to be curative due to the presence of metastases, or tumour microdeposits, that remain undetected at the time of surgery [2], forming a rationale for adjuvant systemic therapies. However, even these treatments have a low cure rate for most solid malignancies that have metastasised. An exception is proving to be melanoma [3], where adjuvant immunotherapy even in the face of metastatic disease can lead to apparent cure. Immunotherapy is in clinical trials for many other cancers. Potentially immunotherapy administered after surgery could stimulate the anti-tumour immune response and control small persisting tumour deposits that lead to disease recurrence. However, tumour draining lymph nodes are often resected for disease staging, and as they are the major repository for anti-tumour T cells, their removal may potentially impact subsequent disease and response to immunotherapy. This study investigates lymph node resection and immunotherapy in a clinically relevant model of lung metastatic disease.

During cancer surgery, the dLNs are removed for staging purposes. The extent of nodal dissection in NSCLC resection is still debated, but it does provide accurate staging information for prognostic purposes, determining the need for adjuvant therapy and confirms complete resection. Indeed, the 8th edition TNM recommends that for NSCLC regional LN involvement be assessed by the removal and pathology on a minimum 6 nodes/stations, 3 from the mediastinum, including the subcarinal node (#7) and 3 from N1 zones [4]. In this process, LNs that do *not* contain tumour cells are usually removed along with any involved ones. Many murine studies by our group [5–8] and by others [9, 10] have shown that *the main site for generation of tumour-specific CTLs is the lymph node draining the tumour* compared to any other lymphoid site or blood. Further, there is a decline in anti-tumour immunity over 7–10 days post-tumour resection [11], and indeed, when a small amount of tumour remains the generation of effective T memory (Tm) cells is better [12]. However, human clinical studies investigating the role of lymph node resection on patient survival are limited, and the efficacy of adjuvant ICPB therapy is currently under study in multiple clinical trials [13].

In this study, we develop a clinically relevant murine model of surgically resectable, but micrometastatic NSCLC, in which we determine the impact of tumour dLN removal at the time of surgery on the development of metastatic lung disease, and its response to ICPB. More specifically, we identify the tumour dLN for surgical resection by fluorescent IRdye tracking and monitor post-surgery metastatic disease survival. Furthermore, we investigate the efficacy of clinically relevant lung cancer immunotherapies. aCTLA4 and aPD-1, after tumour and dLN resection at the time of surgery.

Findings indicate that complete lymphadenectomy at the time of tumour resection is detrimental to survival from lung metastatic disease, and this can be restored with partial lymph node resection or intervention with aPD-1 immunotherapy.

## Materials and methods

*Tumour cell lines*. The murine mesothelioma cell line AB1 was generated by inoculating crocidolite asbestos intraperitoneally (i.p.) in BALB/c mice [14]. The AB1 cell line was then transfected with influenza hemagglutinin (HA) from the Mt Sinai strain of PR8/34/H1N1 influenza virus to generate the well-characterised AB1-HA cell line [5]. Line-1 M cell line was derived from murine Line-1 lung alveolar adenocarcinoma cell line after excision of lung tumour that had naturally metastasized following subcutaneous tumour growth [15]. The cell lines AB1-HA and Line-1 M were transfected to express the luciferase, cell line AB1-HA_LUC and Line-1M_LUC [16]. Cell lines were maintained in RPMI 1640, 20 mM HEPES, 0.05 mM 2-mercaptoethanol, 100 units/mL benzylpenicillin (CSL), 50 μg/mL gentamicin, 10% NCS (Life Technologies) and 50 mg/mL of geneticin for AB1-HA (G418; Life Technologies).

*Tumour metastatic disease models*. Mice received 5 × 10^5^ AB1-HA cells subcutaneously (s.c.) on day 0, tumour growth was monitored with callipers and tumour surgically resected on day 14. On the day of surgery, mice received intravenous (i.v.) injection of AB1-HA_LUC cells. Lung tumour growth was determined by In Vivo Imaging System (IVIS; IVIS Spectrum, PerkinElmer) after i.p. injection with 100 µl Luciferin (15 mg/ml; Sigma-Aldrich). Mice were imaged for peak bioluminescence to 25 min in a Lumina II Imager (Caliper Life Sciences, Hopkinton, MA, USA). Tumour burden was measured as bioluminescence, quantified as photon flux (photons/second), where the limit of sensitivity was considered 0.25 × 10^7^ p/s. Mice were euthanised 88 days post-surgery, or according to clinical signs of severity score sheet, or when tumour burden exceeded 1 × 10^8^ photons/second.

Similarly, for the naturally metastatic Line-1M_LUC cell line, mice received 1 × 10^6^ cells s.c. on day 0, tumour growth was monitored with callipers, and tumour surgically resected at ≥ 140mm^2^. Lung tumour growth was then determined by IVIS. Mice were euthanised 88 days post-surgery, or according to clinical signs of severity score sheet, or when tumour burden exceeded 1 × 10^8^ photons/second. Upon euthanasia, each mouse was dissected for confirmation of axillary and/or inguinal LN resection.

*Tumour resection*. Analgesic buprenorphine s.c. (0.1 mg/kg) was administered 30 min prior to surgery. Anaesthesia was induced with 4% isoflurane in 100% oxygen and maintained at 2% isoflurane, at a flow rate of 2L/min. Resections were performed via elliptical incision over the subcutaneous tumour, spanning twice the length of the lesion, with ≤ 3 mm lateral margins. Tumour tissue was dissected clear of the adjacent fascia, and wounds were closed with 7-mm wound clips (ABLE Scientific, Western Australia) or uninterrupted sutures (dissolvable 5–0 Vicryl sutures, polyglactin 910, Ethicon Australia). After surgery, mice received 0.05 mg/Kg buprenorphine at 12 and 24 h [16].

For CD8 T cell depletion, mice received 100 µg aCD8 IgG antibodies i.p. at day 4, 8 and 12 post-surgery, and T cell depletion was determined in blood as indicated.

*Image-guided tumour draining lymph node resection*. Shaved mice, bearing s.c. tumours (30–40 mm^2^) were injected intratumourally with 1.5 nmol of IRDye 800CW PEG (LI-COR Biotechnology, Lincoln, NE, USA) in 25 μl of PBS. Anaesthesia was induced (as for surgery), and mice serially imaged using the Cri Maestro 2 multispectral imaging system (Caliper LS, Hopkinton, MA, USA).

Inguinal lymph nodes adjacent to the tumour were identified and resected from fat and fascia in the tumour resection margin at the time of primary tumour resection. Axillary lymph nodes were approached with a 5-mm mid-axillary oblique incision, and pectoral muscles retracted. Each lymph node was delicately teased away from the axillary vessel, fat and fascia. The axillary lymph node wound was closed with 5/0 Vicryl (polyglactin 910, Ethicon) uninterrupted sutures. Buprenorphine (0.05 mg/Kg) was administered at 12 and 24 h, or as required. Mice that did not undergo lymph node resection underwent sham surgery.

*Immunotherapy*. On the day of surgery or treatment, mice were randomly assigned to groups. At days 17, 19 and 21 after surgery, mice received immune checkpoint blocking antibodies (BioXcell, West Lebanon, NH) by i.p. injection in PBS, 100 μl for monotherapy or 200 μl for combination therapy, at the following dose: 100 μg aCTLA-4 (clone 9H10, Syrian Hamster IgG1), or 200 μg aPD-1 (clone RMP1-14, Rat IgG2a), and 100 μg aCD40 (clone FGK4.5, Rat IgG2a). Control mice received PBS alone.

*Histology*. At the end of experiment, lung tissue was fixed in 10% formalin (Amber Scientific, Midvale, WA, Australia), embedded in paraffin, and 750 μm sections prepared for haematoxylin and eosin (H&E) staining. Slides were imaged at 20 × on Aperio ScanScope XT (Leica Biosystems, Mt Waverley, VIC, Australia).

*Preparation of single-cell suspensions*. Single-cell suspensions were prepared from lung upon harvest [17]. In brief, lungs were subject to digestion with type IV collagenase (1.5 mg/mL; Worthington Biochemical, Lakewood, NJ) and with type I DNase (0.1 mg/mL; Sigma). Once digested, samples were centrifuged, washed, filtered through 100-µm filters and resuspended in complete media for culture. Lung tumour outgrowth was then determined at day 7 by IVIS.

*Statistical Analysis*. All analysis was performed using GraphPad Prism version 7 software (GraphPad Software Inc., La Jolla, CA) or SPSS* statistics version 22 (Armonk, NY).

## Results

### Tumour draining lymph node resection decreases lung metastatic disease survival

**Fig. 1:**
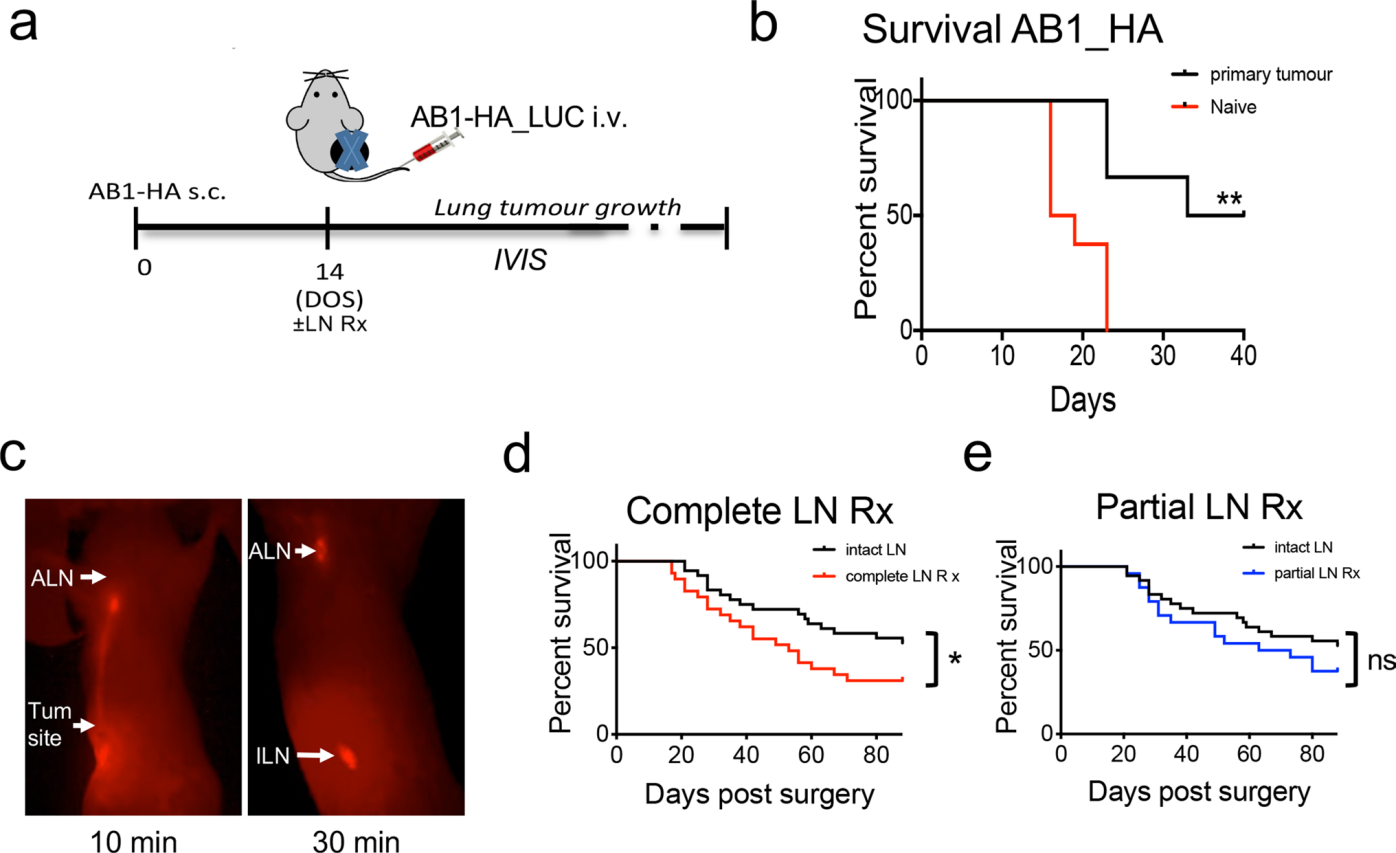
Growth of AB1-HA lung metastases after resection of the primary tumour is partly restrained by a persisting anti-tumour response. (**a**) Experimental schematic, AB1-HA model. Mice received 5 × 10^5^ AB1-HA cells or PBS s.c. on Day 0. On day 14, mice were inoculated with 1 × 10^6^ AB1-HA_LUC cell i.v., and tumours surgically resected. Lymph nodes remained intact, were completely resected, or partially resected, as indicated. Lung tumour growth was measured in the IVIS. (**b**) Kaplan–Meier survival curves post-surgery. (**c**) Lymph node imaging following IRDye® 800CW PEG intratumoural injection on day 14. (ILN = Inguinal LN, ALN = Axillary LN, Tum = tumour). (**d** and **e**) Kaplan–Meier survival curves intact LN compared to complete, and partial, LN resection, respectively. (Combined data from six individual experiments, *n* = 24–36 mice/group). All data were analysed using Mantel–Cox log-rank test; statistical significance given as * = *p* < 0.05, ** = *p* < 0.01, compared to control or ns, not significant

Common lung metastatic disease models involve injection of cells intravenously into healthy mice to seed tumour growth in the lung [18]. As such, the lung metastases develop in mice immunologically naïve to the tumour cells. In the clinical setting however, metastatic disease develops after treatment of the primary tumour, and therefore, the immune system is no longer naïve to the tumour cells. In this study, we developed a new model for metastatic disease that more closely reflects the clinical setting of tumour resection and later onset of metastatic disease. First, we measured the development of lung disease after i.v. tail vein injection in healthy mice, and mice after primary tumour resection (Fig. 1a). All mice in the healthy group had end-stage lung disease by day 23 as determined by IVIS, or clinical signs of severity, and confirmed by histology (Fig. 1b). Median survival was 17.5 days (95%CI) for lung disease in the healthy group. Alternatively, the frequency of lung tumour growth in the primary tumour resection group was 42.8%, with a median survival of 36.5 days (*p* < 0.05; Fig. 1b). This datum indicated a protective effect of primary tumour exposure on subsequent development of metastatic lung disease.

In routine clinical care, patient tumour dLNs are commonly resected at the time of surgery. Therefore, we surgically removed the primary tumour, with or without complete or partial lymphadenectomy and monitored the development of lung metastatic disease (Fig. 1a). To identify the tumour dLNs, we performed intratumoural injection of fluorescent dye (IRDYE® 800CW PEG) to locate ipsilateral axillary and inguinal lymph nodes (Fig. 1c). Notably, removal of the tumour dLNs significantly decreased survival from lung metastatic disease (Fig. 1d; Mantel–Cox log-rank test, *p* = 0.0477). In mice with intact lymph nodes, median survival > 88 days compared to 53 days in the complete LN Rx group (Fig. 1d). Interestingly, with partial LN resection (only the ILN was removed) at tumour resection, there was no change in survival compared to the control tumour resection group (Fig. 1e). This suggests the remaining tumour draining AxLN after surgery provides protection against metastatic disease.

**Fig. 2:**
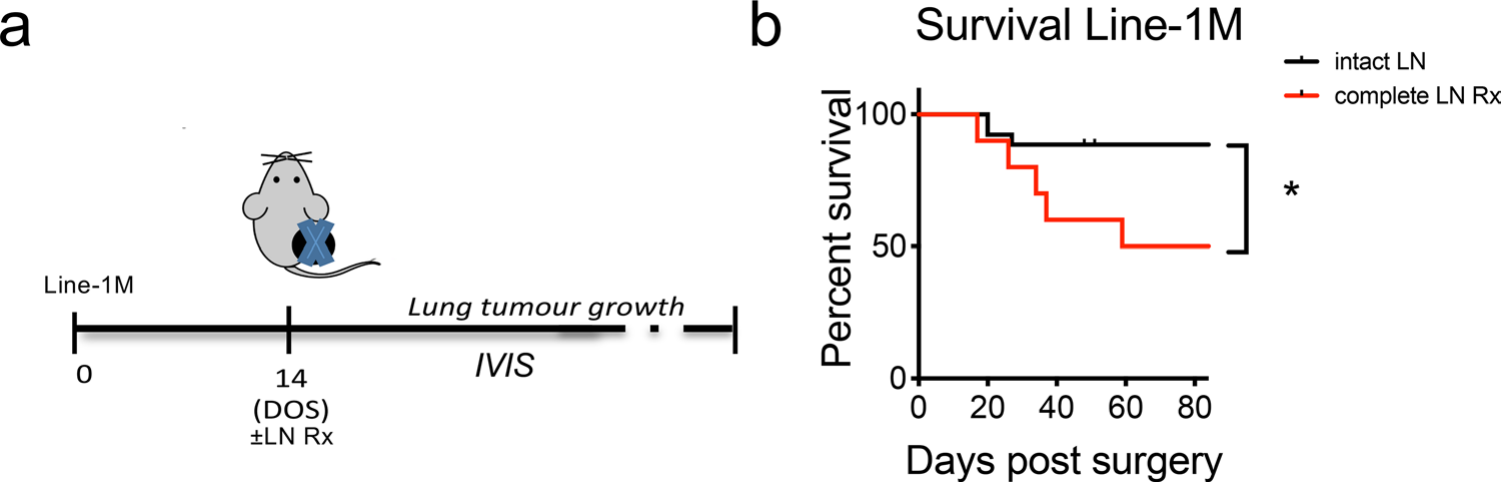
Growth of Line-1 M lung metastases after resection of the primary tumour is partly restrained by a persisting anti-tumour response (**a**) Line-1 M experimental schematic. Mice received 1 × 10^6^ cells s.c., tumours were resected at ≥ 140mm^2^, and development of metastatic lung disease measured by IVIS. (**b**) Line-1 M model Kaplan–Meier survival curve. (Combined data from three individual experiment, *n* = 10–26 mice/group). All data were analysed using Mantel–Cox log-rank test; statistical significance given as * = p < 0.05, compared to control

In order to determine whether there was a similar response to tumour dLN resection in other tumour models, we inoculated mice with Line-1 M adenocarcinoma-derived cell line. In this naturally metastatic disease model, the primary tumour was surgically removed (>140mm^2^), and the onset of lung metastatic disease monitored (Fig. 2a). Complete LN resection significantly reduced survival from lung metastatic disease (log-rank test, *p* = 0.015). Median survival in mice with intact lymph nodes > 88 days compared to median survival of 73.5 days in the complete LN Rx group (Fig. 2b).

These data indicate that complete removal to the dLN at the time of surgery increases the incidence of lung metastatic disease.

### Lung metastatic disease is partially controlled by CD8 T cells

**Fig. 3:**
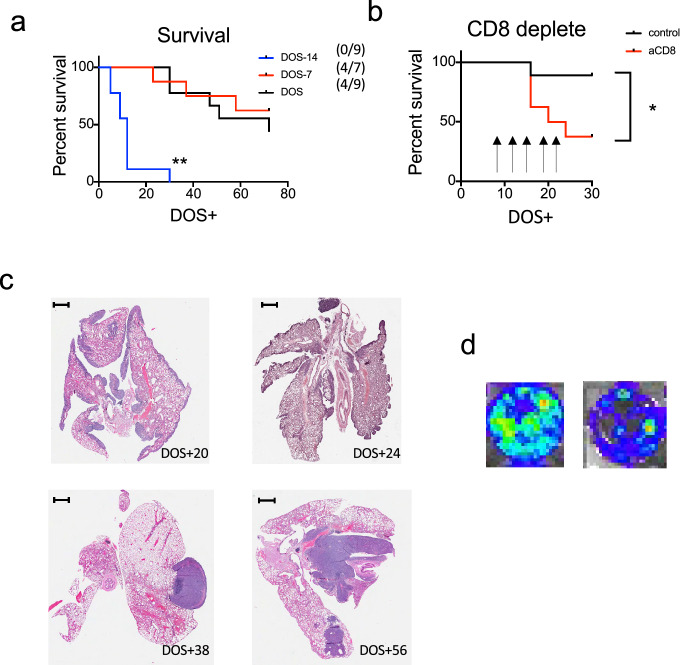
The immunity-generated control of lung metastatic disease is CD8-dependent (**a**) Mice received 5 × 10^5^ AB1-HA cells s.c. on Day 0, and with 1 × 10^6^ AB1-HA_LUC cell i.v. 14 days prior to surgery (DOS-14), 7 days prior to surgery (DOS-7), or on the day of surgery (DOS). (**b**) Mice received 5 × 10^5^ AB1-HA cells s.c. on Day 0 and were inoculated with1 × 10^6^ AB1-HA_LUC cell i.v. on the day of surgery day 14. Mice received anti-CD8 (100 µg/mouse) i.v. on DOS + 6, 10, 13, 18 and 23. Lung tumour growth was measured in the IVIS. (**c**) Mice received 5 × 10^5^ AB1-HA cells s.c. on Day 0, and tumour was resected on day 14. Representative histology (magnification 20x) showing haematoxylin and eosin (H&E) staining of pleural tissues at post-surgery day 20, day 24, day 38 and day 56. (**d**) At day 88, lungs were digested to single-cell suspension and plated in culture, representative plate on IVIS. DOS + , days of post-surgery

In order to determine whether a period of primary tumour exposure is required for protection against metastatic lung disease, we injected AB1-HA i.v. on the day of surgery, 7 days prior to surgery and 14 days prior to surgery. All mice developed lung metastatic disease when i.v. AB1-HA cells were administered on the same day as s.c. tumour inoculation. Whereas nearly half of the mice remained alive at the end of the experiment and survival from lung metastatic disease was significantly longer in mice exposed to subcutaneous AB1-HA tumour growth for a period of 7 or 14 days prior to surgery (Fig. 3a). These data indicate that exposure to the primary tumour for 7 days was protective against the development of metastatic lung disease.

We hypothesised that an immune component derived from primary tumour exposure was able to control occult metastatic tumour cell growth in the lung after surgery. In order to determine an immune component to the protective effect of primary tumour exposure on metastatic disease survival, we depleted CD8 T cells after surgery with aCD8 IgG antibodies in mice (Fig. 3b). CD8 T cell depletion was confirmed in blood by flow cytometry and significantly decreased to 0.41 ± 0.07% at week 1 and 0.26 ± 0.08% at week 2, compared to controls 13.87 ± 0.78% at week 1 and 14.37 ± 0.59% at week 2, respectively (mean ± SE, *p* < 0.001). Strikingly, the survival frequency from metastatic lung disease following post-surgery CD8 T cell depletion was significantly reduced from 88.9% to 37.5% (*p* < 0.05). These data indicate an immunological CD8 T cell component is in part responsible for protection from lung metastatic disease after primary tumour resection.

Next, we examined histological sections from early- and late-onset lung metastases and revealed differing morphology. Early-onset metastasis formed a rind in lung tissue, whereas late metastatic disease manifests as nodules (Fig. 3c). We hypothesised that tumour cells reside in healthy lungs only to develop into metastatic disease. Accordingly, at day 88 post-surgery we digested tissue to single-cell suspension and cultured cells ex vivo for seven days. Notably, all lungs outgrew AB1-HA_LUC cells (Fig. 3d).

These data highlight the role of the immune system in checking the development of lung metastatic disease.

### Adjuvant ICPB response is independent of dLN resection at the time of surgery

**Fig. 4:**
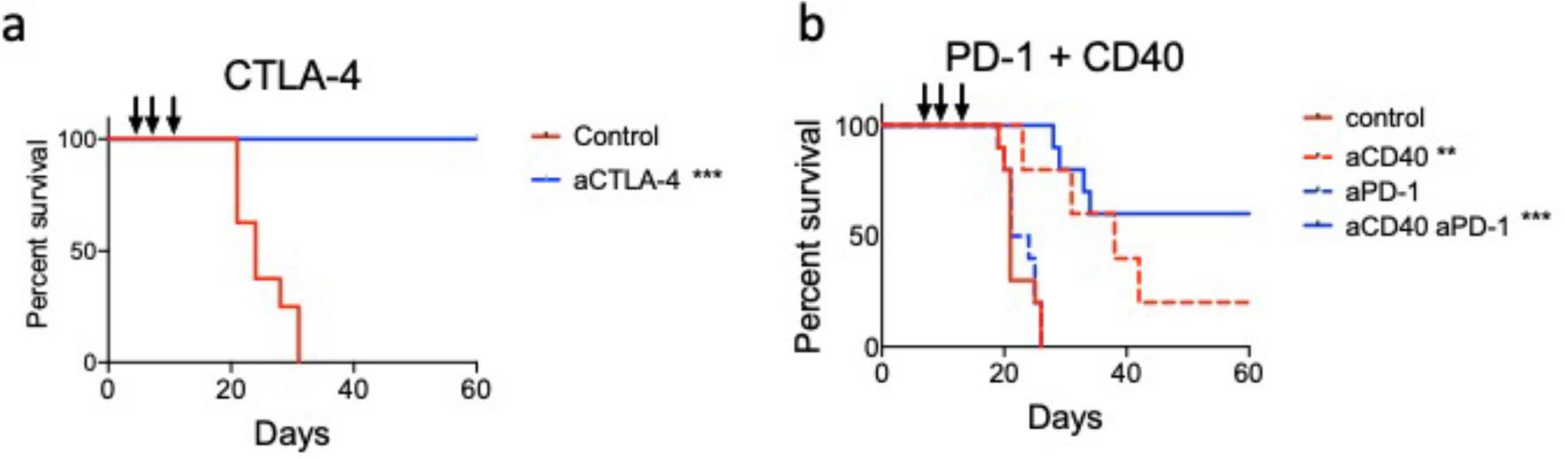
ICPB therapy in the treatment of subcutaneous tumour (**a**) CTLA-4 and (**b**) PD-1 ICPB therapy in AB1-HA primary tumour model. Mice were inoculated s.c. with 5 × 10^5^ AB1-HA cells on day 0, and mice were treated with immunotherapy (100 µg CTLA-4; 200 µg PD-1 and/or 100 µg CD40) on days 10, 12 and 14. Tumour growth was monitored with an end-point of tumour area > 150mm^2.^ Kaplan–Meier survival curves. (Data representative of two or more experiments, *n* = 5–10 mice/group.) All data were analysed using Mantel–Cox log-rank test, ** = *p* < 0.01 compared to control

**Fig. 5:**
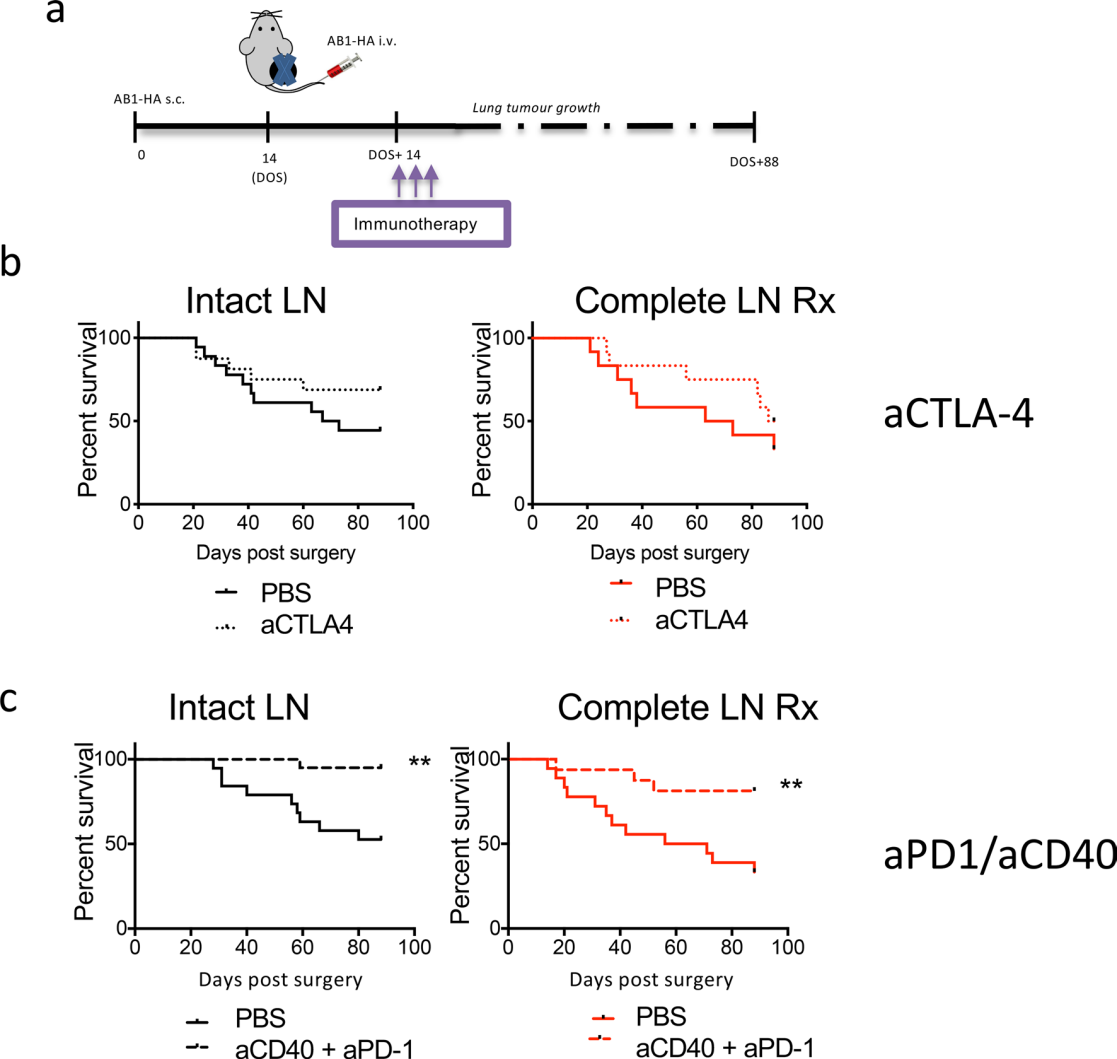
The tumour draining lymph node has a role in controlling metastases but is not required for adjuvant immunotherapy efficacy. (**a**) Metastases immunotherapy model schematic. Mice received 5 × 10^5^ AB1-HA cells s.c. on day 0, and 1 × 10^6^ AB1-HA_LUC cell i.v. on day 14. Tumours were surgically resected from all mice on day 14. Lymph nodes remained intact, or were completely resected, as indicated. At DOS + 14, + 16 and + 18 mice received aCTLA4 ip or aPD1/aCD40 i.p. Lung tumour growth was measured on the IVIS. (**b**) aCTLA4 Kaplan–Meier survival curves. (**c**) aPD1/aCD40 Kaplan–Meier curves. (Combined data of 3–5 individual experiments, *n* = 15–19 mice/group.) All data were analysed using Mantel–Cox log-rank test, ** = *p* < 0.01 compared to control

Given the observed immune component to lung metastatic disease development, we determined the efficacy of checkpoint, aCTLA-4 and aPD-1, on survival in both the primary tumour (Fig. 4) and metastatic disease model with complete dLN resection (Fig. 5a).

The efficacy of immunotherapy in primary tumours was first determined. Treatment of primary subcutaneous tumour with aCTLA4 increased survival (Fig. 4a), in keeping with previous findings^16^. Next, we determined the efficacy of aPD-1 treatment in our subcutaneous primary tumour model (Fig. 4b). aPD-1 treatment did not increase survival from primary tumour, whereas for aCD40 treatment there was 20% survival at day 60. However, there was 55% survival for the aPD-1 treatment group in combination with the co-stimulatory molecule aCD40 [19] (Fig. 4b).

Effective primary tumour treatment strategies, aCTLA4 and aPD-1/CD40, were selected for treatment in the adjuvant setting (schematic, Fig. 5a). Notably, treatment with aCTLA4 post-surgery did not significantly increase survival from lung metastatic disease (Fig. 5b). Median survival in aCTLA4 treated mice with intact lymph nodes (>88 days), and this was not significantly different from the untreated intact LN Rx group (median survival of 72 days). Similarly, there was no significant difference between the complete LN Rx aCTLA-4 treated and untreated groups (>88 days and 73 days, respectively). Alternately, aPD-1/aCD40 immunotherapy post-surgery increased survival from lung metastatic disease (Fig. 4c). Median survival after tumour resection and aPD-1/aCD40 therapy was significantly longer compared to the tumour resection only group (Mantel–Cox log-rank, p = 0.0022). In addition, there was an increased survival in the tumour resection with lymph node removal group treated with aPD-1/aCD40 treatment (> 88 days) compared to untreated (63.5 days, Mantel–Cox log-rank p = 0.0064).

Notably, mouse lungs harvested after checkpoint blockade, aCTLA4 or aPD-1/aCD40 all grew out AB1-HA_LUC cells, regardless of LN resection.

These data indicate that aPD-1/CD40 ICPB therapy after surgery is more effective against lung metastatic disease than aCTLA-4 treatment.

## Discussion

Surgery is the gold standard treatment for many cancers; however, long after surgery patients often develop metastatic disease. This is likely due to occult tumours cells remaining at the time of surgery. Cancer surgery generally incorporates lymph node resection, for disease staging; however, this also potentially removes vital anti-tumour immune surveillance mechanisms required for effective control of local and metastatic disease. It is reasonable to consider that LN resection may impact on immunotherapy treatments that aim to modulate systemic anti-tumour immune cell responses. Thus, an understanding of the implications of lymph node resection for metastatic disease and response to immunotherapy is required. This study investigated onset of metastatic disease, the impact of lymph node resection, and efficacy of adjuvant immunotherapy in a novel pre-clinical model of lung metastatic disease that closely reflects the clinical setting.

The ability of cancer cells to remain dormant for extended periods of time post-surgery has long been thought due to a state of equilibrium between anti-tumour immune cells and the cancer cells [20]. Notably we further determined in this study, where lung metastatic disease developed at a clinically relevant site where approximately half of all subjects developed metastatic disease, and this was demonstrated as at least partially due to immune responses to the primary tumour. Previous data in the AB1-HA subcutaneous tumour models have indicated that a very small number of tumour cells reside in the primary tumour draining lymph nodes but are hard to find. Whilst they did not manifest as disease in vivo, the tumour cells could be grown ex vivo from lymph nodes [7]. In this study, we observed that early lung metastatic disease emerged as rinds in the lung periphery, whilst late-onset metastatic disease formed tumour nodules, potentially reflecting tumour cell escape from immunosuppression in the latter. An extension to this work would be further investigation in orthotopic lung cancer models; however, one of the many strengths of our model is that the tumours are precisely measurable and able to be resected, whereas surgical resection in the orthotopic model is difficult if not impossible in the experiments undertaken in this study.

We, and others, have shown that tumour dLNs have been shown to harbour vital tumour-specific T cells [5, 7, 8, 16, 17, 21, 22]. Notably, after partial tumour resection tumour antigen presentation continues in the dLN for 7 to 14 days [7] driving the development of CD8 T effector memory cell responses to reduce local residual disease or protect against tumour development on re-challenge [11, 12]. However, there are very few studies determining the role of the lymph node in the metastatic disease setting. In our pre-clinical lung metastatic disease model, the frequency of survival was reduced on complete lymph node resection at the time of surgery, and notably survival was restored with partial lymph node resection. These data are highly relevant to the clinical setting in which complete lymph node resection is routinely performed for disease staging. Our data suggest that partial lymph node resection may be advantageous, leaving tumour-free lymph nodes intact, to retain anti-tumour immune cells that can fight cancer cells and increase patient survival from metastatic disease.

Adjuvant ICPB is under investigation in a number of clinical trials, and it is possible that lymphadenectomy may impact immunotherapy treatment outcomes. Recent studies have indicated that the tumour-specific T cell response in the tumour dLN closely correlates with response to ICPB [21]. Surprisingly, complete dLN resection did not alter lung metastatic disease survival frequency for either aCTLA4 or aPD-1/CD40 treatment. In this study, in our model of metastatic disease aCTLA4 treatment did not improve survival. Alternatively, treatment with aPD-1/aCD40 therapy was effective and increased survival from metastatic lung disease. Interestingly, this reflects the findings for aCTLA4 treatment in lung cancer patients and malignant mesothelioma, in which a single agent, ipilimumab and tremelimumab have demonstrated poor efficacy [13, 23, 24]. Alternatively, nivolumab, and pembrolizumab have FDA approval for the treatment of metastatic lung cancer patients [13].

Notably, CTLA-4 is a homologue of CD28 and a negative regulator of T cell function via modulation CD80 and CD86 response [13]. Draining lymph nodes may have their dominant role early in the immune process, but after the tumours are established that may not be so crucial. The observed lack of efficacy of aCTLA4 may reflect mode of action, which is early in the T cell response, and accordingly it may be more effective in the treatment of primary tumours but not for later metastatic disease. Alternatively, as the expression of CTLA-4 is also high on Treg cells, leading to T cell immunosuppression [25], an effect on these cells may also be present. The role of Treg tumour suppression within lung tissue remains to be investigated. Alternatively, PD-1 ligand engagement abrogates T cell signalling to essentially reactivate an exhausted T cell phenotype [23, 26], unleashing T cells to drive effective anti-tumour immune responses. This work highlights that effective tumour control in the metastatic disease setting is due to complex immune cell to tumour cell interactions that are likely time dependent. Importantly, the finding of efficacy for aPD-1/aCD40 after lymph node resection has direct clinical impact and provides urgency for early intervention strategies to be enacted after surgery to ameliorate metastatic disease onset.

The success of adjuvant immunotherapy is apparent in findings from clinical trials; however, efficacy for murine primary tumour models has often not translated to patient studies. For example, aCTLA4 is effective in the treatment of solid subcutaneous tumour in mice [17, 27], but is not effective in NSCLC clinical trials. Further, a plethora of ICPB treatment strategies that have shown efficacy in subcutaneous tumour models are currently in clinical trials. This includes combination of aCTLA-4 and aPD-1 (CheckMate 012 Study, CheckMate 568), aOX40 therapy (NCT02315066), aPD-1/TIM-3 bi-specific antibodies (NCT03708328), aCD40 (APX005M) + nivolumab, combination aCTLA4/aPD1or PDL1/chemotherapy strategies (NCT03515629;NCT02279732), and many others still awaiting clinical trials. In order to improve the success of NSCLC clinical trials, better models for pre-clinical immunotherapy screening are required, and this could potentially include investigation in our model of surgery and lung metastatic disease.

In summary, we established the model as described to provide a precise scientific method of evaluation because animals, like humans, generate metastases unpredictably. We clearly show a decrease in lung metastatic disease survival following complete lymph node resection, with restoration of survival with conservative lymph node resection in two pre-clinical metastatic disease models. In the AB1-HA metastatic disease model, with controlled systemic introduction of tumour cells at the time of surgery, lymph node retention decreased metastatic disease, implicating immune control of introduced tumour cells. Importantly, notwithstanding the unpredictability of natural metastases in animal models, we did show similar findings of increased survival from metastatic disease were observed in the naturally metastatic, though capricious disease model Line-1 M. In addition, the study highlights the need to test ICPB in relevant clinical models. As such, aCTLA4 therapy was effective in subcutaneous solid tumours but lacked efficacy in the post-surgery metastatic disease setting. Alternately, ICPB treatment with aPD-1/aCD40 was efficacious in both the subcutaneous solid tumour and metastatic disease models of disease. Finally, frequency of survival following aPD-1/aCD40 therapy was similar with or without lymphadenectomy. This study provides valuable information in a relevant pre-clinical model of metastatic disease, demonstrating the impact of lymph node resection, and immune checkpoint blockade response after surgery on survival. Furthermore, the work highlights the importance for suitable pre-clinical modelling to facilitate clinical translation.

Finally, there is a paucity of information regarding the impact of lymph node resection in development of metastatic disease and/or response to ICPB, and this reflects the difficulty to address this in patient’ studies. This study is informative to surgeons and addresses many questions relevant to the impact of lymph node resection, recommended by the AJCC Cancer Staging Manual, during primary tumour surgery relevant to patient survival and response to immunotherapy.

## Declarations

### Animal Source

Eight-week-old female BALB/c mice were obtained from the Animal Resource Centre (Murdoch, WA, Australia).
