# Correction to: Tumour draining lymph node-generated CD8 T cells play a role in controlling lung metastases after a primary tumour is removed but not when adjuvant immunotherapy is used

**DOI:** 10.1007/s00262-021-02970-z

**Published:** 2021-06-05

**Authors:** Vanessa S. Fear, Catherine A. Forbes, Samuel A. Neeve, Scott A. Fisher, Jonathan Chee, Jason Waithman, Shao Kang Ma, Richard Lake, Anna K. Nowak, Jenette Creaney, Matthew D. Brown, Christobel Saunders, Bruce W. S. Robinson

**Affiliations:** 1 Institute for Respiratory Health, National Centre for Asbestos Related Diseases, University of Western Australia, Perth, Australia; 2 Telethon Kids Institute, Perth, Australia; 3 Medical School, School of Biomedical Sciences, University of Western Australia, Crawley, WA Australia; 4 Fiona Stanley Hospital, Murdoch, Australia; 5 Division of Surgery, Medical School, University of Western Australia, Perth, Australia

## Correction to: Cancer Immunology, Immunotherapy 10.1007/s00262-021-02934-3

The original version of this article unfortunately contained a mistake. The given name and family name of authors were swapped.

The correct given name and family name should be:

Vanessa S. Fear

Catherine A. Forbes

Samuel A. Neeve

Scott A. Fisher

Jonathan Chee

Jason Waithman

Shao Kang Ma

Richard Lake

Anna K. Nowak

Jenette Creaney

Matthew D. Brown

Christobel Saunders

Bruce W. S. Robinson

The original article has been corrected.

